# Quality of life in children with unilateral hearing loss undergoing cochlear implantation: A systematic review and meta-analysis

**DOI:** 10.1016/j.bjorl.2025.101628

**Published:** 2025-05-14

**Authors:** Paola Piva de Freitas, Rafael Freire de Castro, Ana Paula de Morais e Oliveira, Carlos Takahiro Chone, Arthur Menino Castilho

**Affiliations:** a Universidade Estadual de Campinas (Unicamp), Faculdade de Medicina Ciências (FCM), Departamento de Otorrinolaringologia e Cirurgia de Cabeça e Pescoço, Campinas, SP, Brazil; b Universidade Estadual de Campinas (Unicamp), Faculdade de Medicina Ciências (FCM), Biblioteca, Campinas, SP, Brazil

**Keywords:** Unilateral hearing loss, Cochlear implant, Quality of life, Child

## Abstract

**Objectives:**

Children with Single-Sided Deafness (SSD) may experience a range of developmental challenges. While cochlear implantation has demonstrated significant improvements in hearing, its impact on Quality of Life (QoL) remains underreported. This review evaluates how cochlear implantation for SSD influences the quality of life in children.

**Methods:**

A systematic review was conducted following the PRISMA guidelines across nine different databases, without language restrictions, including articles indexed up to December 15, 2023. Eligible studies included patients up to 18-years-old; severe to profound unilateral hearing loss, and normal contralateral hearing; cochlear implantation with more than 3 months of follow-up; QoL assessed by structured questionnaires. Meta-analysis compared QoL scores obtained between pre- and post-operative periods for time of onset or hearing loss (congenital vs. post-lingual), evaluated by parents and children, and the effective cochlear implant usage time.

**Results:**

A total of 296 articles were identified, with 6 eligible for qualitative analysis and 3 for meta-analysis, involving 187 patients. The mean age at implantation was 5.8 years, with an average auditory deprivation time of 3.8 years. Both the congenital and post-lingual groups demonstrated improvement in QoL, with a mean increase of 1.51 points in children's evaluations (*p*-value < 0.001) and 2.70 points in parental perspectives (*p*-value < 0.001), assessed on a 10-point scale. The estimated effective device use time was 8.8 h per day, with 8.55 h per day for the congenital group and 10.37 h per day for the post-lingual group. There was no statistically significant difference in usage time between the two groups (*p*-value = 0.140).

**Conclusion:**

The results indicated a significant improvement in QoL, as reported by both parents and through self-assessment. The treatment also demonstrated high levels of adherence. Both congenital and post-lingual groups yielded similar outcomes in terms of QoL and device usage time.

## Highlights

• Children with SSD experience many developmental challenges and poor quality of life.
• CI has previously been shown to improve hearing, although QoL outcomes remain underreported.
• This review shows improvement in QoL for parents and children and good adherence to CI.
• Similar QoL improvement and duration of use for congenital and post-lingual groups.
• Factors associated with discontinuation need further investigation.

## Introduction

Single-Sided Deafness (SSD) has long been recognized in audiology, but it was not classified as a pathology until the 1940s when its damage was understood.^1^ First studied in adults,^2^ it was identified in children in the 1980s.^3^, ^4^, ^5^ Advances in electrophysiological testing and neonatal hearing screening programs have led to earlier diagnosis,^6^, ^7^ and this has led to discussions about the best method of rehabilitation and timing of intervention. However, these questions have not been fully answered in clinical practice. This study evaluates the Quality of Life (QoL) of children with SSD undergoing Cochlear Implantation (CI) and correlates it with the time of installation of the hearing loss and the perspective (parent vs. child). In addition, the usability of the device and its correlations with the time of installation of the hearing loss will be evaluated.

SSD is defined as severe to profound unilateral hearing loss with normal hearing in the contralateral ear. Using pure-tone audiometry, SSD was identified when the average of pure-tone air conduction thresholds at 0.5, 1, 2 and 4 kHz (PT4) in one ear was ≥70 dB Hearing Level (HL) with ≤20–30 dB HL in the contralateral ear, depending on the source used^8^, ^9^, ^10^ It is a rare condition with an estimated incidence of 0.6 to 0.7 per 1000 births^9^, ^11^ Recently, Dewyer et al. analyzed audiometric data from more than 50,000 children with a median age of 7-years and found a prevalence of 0.36%.^8^

Different pathophysiological mechanisms lead to SSD with variable incidence depending on the age of onset. Because of the close correlation between hearing and language development, hearing losses are often categorized according to the time of onset versus the expected age for this milestone in language development. Arndt et al.^12^ classified etiologies as congenital, perilingual (defined as >0–4 years of age) and post-lingual (defined as >4 years of age). The same author studied a cohort of 50 children with SSD and found a higher proportion of congenital/perilingual cases (n = 30), with almost 50% of these cases having cochlear nerve malformations (aplasia or hypoplasia), followed by unknown causes and Cytomegalovirus (CMV) infection.^13^

Other causes such as meningitis and cranioencephalic trauma started to occur in the perilingual group, while Enlarged Vestibular Aqueduct (EVA) was the most common cause in the post-linguals.^13^

### Physiology of binaural hearing

Binaural hearing adds redundancy and integration to the afferent stimuli, providing advantages in sound source localization, speech intelligibility in noisy environments, and reduced auditory effort with better and more accurate information processing.

In situations where the sound of interest and the background noise are spatially separated, there is a mechanical hearing benefit known as the head shadow effect. For example, if the sound is presented on the right and the distracting noise is presented on the left, the head provides an acoustic shadow that attenuates the arrival of the noise in the right ear, resulting in an improvement in the signal-to-noise ratio in the right ear. This effect is purely physical, and not due to central integration and processing, although the brain automatically tries to direct attention to the ear with the best signal-to-noise ratio to take advantage of this phenomenon, resulting in better intelligibility.^14^

Due to the sound wave nature, frequencies are affected differently by the head shadow effect. Higher frequency sounds, particularly those above 1500 Hz, have a shorter wavelength relative to the size of the head and are more attenuated than low frequency sounds. High frequencies are attenuated by approximately 20 dB, while low frequencies are attenuated by 3- to 6-dB.^14^

Another mechanism favored by the spatial separation between the sound source and the noise generator is the Squelch Effect. Unlike the head shadow effect, this involves central processing, where the target sound is amplified while the noise is masked, enhancing the signal-to-noise ratio.^15^, ^16^ This phenomenon facilitates discernment of subtle differences in intensity and frequency, improving speech recognition in noise.^17^ This effect can increase speech intelligibility by 2–4.9 dB.^18^

As a result of the bilateral afference, there is also the effect of Binaural Summation, where the sum of the auditory stimuli from each ear reaches the brainstem more intensely, giving the impression of increased sound intensity. This effect yields a gain of 2- to 3-dB^18^ and can reach up to 10 dB in some literature.^17^ It also increases sensitivity to distinguish different intensities and frequencies, improving speech understanding in both noisy and quiet environments.^16^ Like the Squelch effect, Binaural Summation is the result of central auditory processing, but unlike the former, it is thought to occur when both ears are exposed to a similar signal.^16^

Binaural hearing also aids in sound source localization. The central integration of information, especially the difference in time and intensity that the sound reaches each ear, helps determine spatial orientation. In situations where the sound is presented laterally and in a horizontal plane, the individual's head acts as a shield for the sound wave, so the stimulus arrives at a lower intensity and relatively late in the ear that is contralateral to the sound source^15^ The interaural time difference in humans varies from 0 to 700 μs, with a difference of 10 μs already being perceived.^17^ Its effect is more important in the identification of lower frequencies, especially up to 1000 Hz, because to the length of the sound wave is proportionally greater than the size of the head, which produces less attenuation of the wave.^15^ At higher frequencies, and therefore at shorter wavelengths, the difference in interaural intensity is a better cue to the location of the sound.^15^, ^18^ Identification in the vertical plane also adds to these effects the diffraction of sound caused by the anatomy of the ear and the position of the head.^15^, ^17^

### The impact of unilateral hearing loss on children

SSD influences cortical development and plasticity^19^ promoting adaptive central reorganization favoring the normal ear over the impaired ear, known as Aural Preference Syndrome.^20^, ^21^ It also acts by compromising binaural integration, expressed by a reduction in sensitivity to binaural stimuli.^22^, ^23^

In patients with SSD, especially when it is congenital or prelingual, these changes in the maturation of the auditory pathways also generate alterations in the brain networks associated with higher cognitive functions, impacting various aspects of child growth and development,^18^, ^24^, ^25^ notably: Impaired speech recognition in noise;^26^, ^27^ Reduced localization skills;^27^, ^28^, ^29^ Speech and language delas;^26^, ^30^, ^31^, ^32^, ^33^ School developmental disabilities;^30^, ^34^, ^35^ Balance deficits;^36^ Behavioral changes;^34^ Cognitive déficits;^30^, ^37^, ^38^ Low quality of life.^39^, ^40^

### Therapeutic modalities

Therapeutic modalities for sound redirection, such as hearing aids with the CROS system and bone anchored prostheses, are based on the sound transmission to the normal hearing ear, reducing the shadow effect of the head, improving the signal-to-noise ratio and consequently sound perception.^41^ They are alternatives used in the adults but generally avoided in children due several reasons: the immaturity of their auditory system, which hinders device and environmental management, impacting device performance;^11^, ^42^ The necessity to occlude the normal ear with an earmold, potentially limiting access to sound and consequently impact the natural hearing development in that ear;^42^ the inability to prevent cortical changes associated with auditory deprivation, thus not truly restoring binaural hearing;^11^ and low adherence to the method, even when a hearing improvement is observed.^43^

In this context, CI is the only alternative capable of restoring binaural hearing and has emerged as an important therapeutic alternative.^41^

### Cochlear implantation × binaural hearing

Response to CI in pediatric patients with SSD shows benefits in sound source localization,^13^, ^16^, ^44^, ^45^ speech perception in noise^16^, ^45^, ^46^, ^47^ and improved quality of life^12^, ^46^, ^48^, ^49^, ^50^, ^51^, ^52^ and that these benefits persist over time.^53^ CI, particularly when performed early, has been shown to potentially prevent^54^ or even partially reverse changes in the auditory cortex caused by hearing deprivation.^19^ In addition to the audiological gains, CI has been associated to enhanced language skills by Arras et al.^55^, ^56^ They followed children with SSD who underwent CI at a prelingual age and compared them with two control groups: normal hearing and prelingual SSD who did not receive CI. The author described consonant performance between the implanted patients and their normal-hearing peers, while the non-implanted group showed poorer performance in initial grammatical acquisition (at 2–3 years of age)^55^ and in later narrative skills, including verbal short-term memory and narrative abilities (at 4–6 years of age).^56^

The CI was recently approved in the United States for treating children with SSD over age 5. MedEl Corporation (MED-EL GmbH, Innsbruck, Austria) received clearance first, in 2019, followed by Cochlear Corporation (Cochlear Americas Corporation, Sydney, Australia) in 2022.^11^

While the optimal timing for implantation in children with SSD remains uncertain, research in other pediatric populations with hearing loss who underwent CI early suggests better outcomes in speech, language, and functional abilities.^57^ Furthermore, surgical risks are not higher when performed in patients under 1 year of age.^58^ Recently, Patro et al.^59^ evaluated 19 children with SSD, implanted at a median age of 2.8 years. They found that these children demonstrated good speech recognition, consistent device use, and no postoperative complications.

Despite the current evidence supporting the benefits of rehabilitation, the establishment of a new treatment regimen for a child with an invisible impairment continue to create resistance within the medical community. Consequently, studies on the subjective aspects of rehabilitation are becoming increasingly important.

## Methods

This systematic literature review followed the Preferred Reporting Items for Systematic Reviews in Meta-Analyses (PRISMA) guidelines and was registered on the International Prospective Register of Systematic Reviews (PROSPERO) platform with registration number CRD42024503680.

### Search strategy

The searches were conducted across multiple databases including MEDLINE/PubMed, PMC, Web of Science, Scopus, Embase, Cochrane Collaboration Library, Biblioteca Virtual em Saúde/Bireme, Proquest and EbscoHost databases, with the last update performed on December 15, 2023. There were no time or language restrictions.

In MEDLINE/PubMed the search strategy employed was: "(Infant OR "Child, Preschool" OR Child OR Adolescent) AND ("Hearing Loss, Unilateral" OR"single-sided deafness" OR "Single-sided deafness (SSD)" OR "single-sided deafness" OR "unilateral sensorineural hearing loss") OR (Deafness OR "Hearing Loss") AND (monoaural OR monaural OR "monaural hearing") AND "Cochlear Implantation" AND ("Quality of Life" OR "Psychosocial Impact")".

### Selection criteria

The eligibility criteria were applied following the PICOS strategy:

1 Patients: Children up to 18-years old with unilateral severe to profound hearing loss and normal hearing in the contralateral ear. Normal hearing was defined as PT4 audiometric thresholds ≤20 dBHL. Severe to profound hearing loss was defined as PT4 audiometric thresholds ≥70 dBHL.
2 Intervention: Cochlear implant surgery with a minimum of three months of postoperative follow-up.
3 Controls: Patients themselves, in the preoperative period, without any rehabilitation. Studies utilizing alternative forms of control were only included in the qualitative analysis.
4 Outcome: Quality of life measured by structured questionnaires, both self-administered and answered by parents.
5 Study design: Experimental and epidemiologic studies were included, including randomized clinical trials, nonrandomized clinical trials, quasi-experiments, before-and-after studies, cohort studies, and case-control studies.

Studies that combined data from adults and children without a clear distinction between the groups; studies that overlapped previously analyzed cohorts; systematic reviews, opinion articles, editorials, conference abstracts, and research protocols; case reports with samples of less than 5 patients were excluded.

### Data extraction and quality assessment

The studies were imported into the EndNote and Rayyan platforms, where duplicates were excluded. Two authors (PF and RC) independently and blindly assessed the titles and abstracts against the eligibility criteria. Studies meeting the criteria or those uncertain were obtained for full analysis. A subsequent independent and blinded selection was conducted. Discrepancies were resolved through a consensus meeting, involving a third senior reviewer (AC) when necessary.

The methodological quality of the studies was evaluated using the Newcastle-Ottawa Scale,^60^ adapted for this review's specific needs. Studies scoring less than six points were discussed by the authors regarding their inclusion in the review.

The quantitative data was extracted using the WebPlotDigitizer program, version 4.6.^61^ The authors of the selected studies were contacted to provide data for qualitative analysis, but no response was received by the time the data analysis was completed.

The dataset included demographic information, age of hearing loss onset, etiology, age at implantation, duration of hearing deprivation, length of effective cochlear implant use, and QoL questionnaire results.

The classification proposed by Arndt et al.^12^ was adopted in this study to allow comparisons between studies, categorizing the time of onset of hearing loss as congenital (diagnosis at birth), perilingual (diagnosis between 0 and 4 years of age), and post-lingual (diagnosis after 4 years of age).

The Speech, Spatial and Qualities of Hearing Scale (SSQ) questionnaire,^62^ in its caregiver-administered and patient self-reported versions, was used to assess QoL in a standardized manner. This approach facilitated direct comparisons of results across studies and reduced the risk of bias. The SSQ provides a subjective assessment of hearing ability through three general domains: speech (in quiet, on the telephone, in groups and/or in noisy or reverberant environments), spatial hearing (the location and direction of sounds), and other qualities of hearing (including recognition and segregation of sounds, ease of listening, sound identifiability, and naturalness/clarity). It utilizes a 10-point scale, presented as a ruler, for respondents to rate their experience in the described scenarios, with higher grades related to better performance in the situation. The version for children includes 33 questions, while the parental version contains 23. This questionnaire is recognized for its ability to evaluate dynamic binaural hearing functions.^62^, ^63^, ^64^

For studies with overlapping populations.^12^, ^13^, ^16^, ^50^^,^^65^, ^66^, ^67^ only the most recent publication was included.

### Statistical analysis

The statistical analyses were performed using *R* software, version 4.3.0. Only studies comparable in terms of design, outcomes, and the applied questionnaire were included in the meta-analysis. Studies deemed unsuitable due to heterogeneity or risk of bias were analyzed individually through a descriptive review.

In this review, random-effects models were employed to estimate the difference between the two assessment times (preoperative and postoperative) using the Restricted Maximum Likelihood (REML) method. The *Q* test was employed to identify heterogeneity among the studies, while the I² statistic was used to quantify it. This statistic estimates the proportion of observed heterogeneity across studies, ranging from 0% to 100%; higher values indicate greater differences between studies. A significance level of 5% was adopted.

## Results

**Fig. 1:**
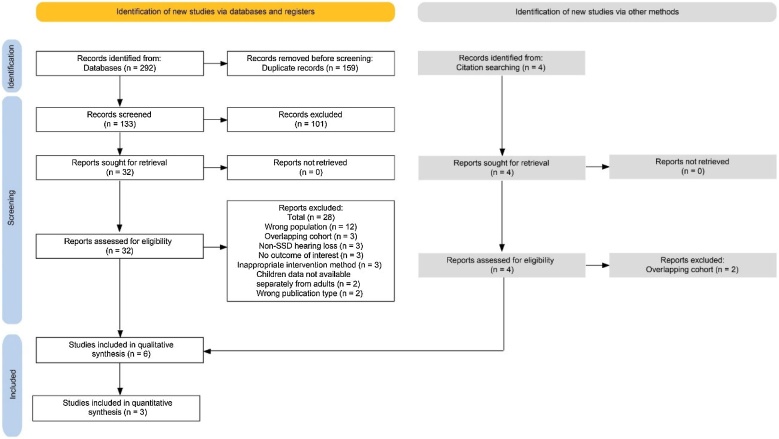
PRISMA flowchart.^70^

A total of 296 articles were assessed, with 292 retrieved from databases and 4 obtained through reference and citation searches. After excluding duplicates (159), screening titles and abstracts (101), evaluating full-text eligibility criteria (28) and identifying overlapping cohorts (2), 6 articles, published between 2017 and 2023, were selected. Of these, 3 studies were included in the meta-analysis (Fig. 1).

Study sample sizes ranged from 19 to 57, totaling 187 individuals (Table 1). Regarding the onset of hearing loss, 106 subjects were classified as congenital, 30 as perilingual, 45 as post-lingual, and 6 remained unidentified. The children were implanted at a mean age of 5.8 years, with a mean duration of hearing loss of 3.8 years. The mean follow-up period was also 3.8 years, and the mean time of effective use of the implant was 8.8 h/day. The meta-analysis excluded the studies by Hicks et al.^50^ due to the absence of individual QoL data categorized by the time of onset of hearing loss; Gordon et al.^51^ because they did not provide individual comparisons of QoL before and after surgery; and Zeitler et al.^49^ because they employed a different QoL instrument, rendering comparisons prone to bias.

**Table 1:** Characteristics of the included studies: qualitative analysis.

						Years, Mean (standard deviation) [range]					
Source, year	Location	Period	Study design	Nº of Patients	Male	Age at Implantation	Time of hearing deprivation	Etiology	Questionnaire	Comparator	Follow-up, years
Arndt et al., 2023^a^	Germany, Freiburg	2009‒2021	Cohort, retrospective	36	58.3%	6.1 (4.5) [1–16.6]	3.8 (4.0) [0–16.6]	10 idiopathic	SSQ for parents and children	Before and after comparison	4.8 (2.7) [1.7–11.8]
								9 CMV			
								3 trauma			
								2 recurrent otitis	HEAR‒QL		
								2 sudden deafness			
								2 malformation (EVA)			
								1 other malformations	IOI‒HA		
								1 meningitis			
								1 perinatal hypoxia			
								1 cholesteatoma	KINDL		
								1 auditory neuropathy			
								1 ototoxicity			
								1 labyrinthitis			
								1 cochlear nerve dysplasia			
Zeitler et al., 2023	United States, multicenter	Not available	Transversal	31	41.9%	6.73 (3.17) in the SSD group	6.76	14 idiopathic	Children with Cochlear Implants: Parental Perspectives Survey	Children undergoing bilateral CI	N/A
								9 evaluation unknown			
								5 malformation (EVA)			
								2 CMV			
								1 genetic (Noonan syndrome)			
Hicks et al., 2023	United States, North Carolina	Not available	Cohort, Prospective	19	57.8%	5.2 (1.2)	3.4 (1.7)	11 Idiopathic	SSQ for parents	Before and after comparison	2
								3 CMV			
								2 malformation (IP‒II)			
								1 infection			
								1 trauma			
								1 genetic (Waardenburg syndrome)			
Gordon et al., 2023	Canada, Toronto	2013‒2021	Cohort, Prospective	57	Not available	2.47 (1.58) prelingual group	1.93 (1.56)	21 CMV	SSQ for parents and children	Children with early versus late onset SSD	6.6
								15 idiopathic			
								9 genetics			
								5 meningitis			
						11.67 (3.91) postlingual group		4 trauma			
								2 cholesteatoma			
								1 LSCC erosion			
Marcías et al., 2019^a^	Spain, Las Palmas of Gran Camaria	2013‒2016	Transversal	23	47.8%	6 (2.4) [0.9–9.9]	1.27 (0.84) [0.9–4.1]	4 congenital	SSQ for parents	Before and after comparison	N/A
								19 acquired			
Thomas et al., 2017^a^	Germany, Bochum	2012‒2016	Cohort, retrospective	21	38%	5.6 (3) [0.8–11.3]	Same as implantation (evaluated only congenital cases)	12 CMV	SSQ for parents and children Custom questionnaire	Before and after comparison	1.9 (1.3) [1.1–3.7)
								2 Rubella			
								1 malformation (EVA)			
								1 genetics			
								1 premature birth			
								1 family history of hearing loss			

CMV, Cytomegalovirus; EVA, Enlarged Vestibular Aqueduct; SSD, Single-Sided Deafness; LSCC, Lateral Semicircular Canal; N/A, Not Applicable.

a Considered in the Meta-analysis.

To assess the impact of implant use and the timing of hearing loss onset on quality of life, a meta-regression analysis was conducted with time (pre- vs. postoperative) and hearing loss onset (congenital/perilingual vs. post-lingual) as factors. Separate models were created for children and parents’ responses.

**Fig. 2:**
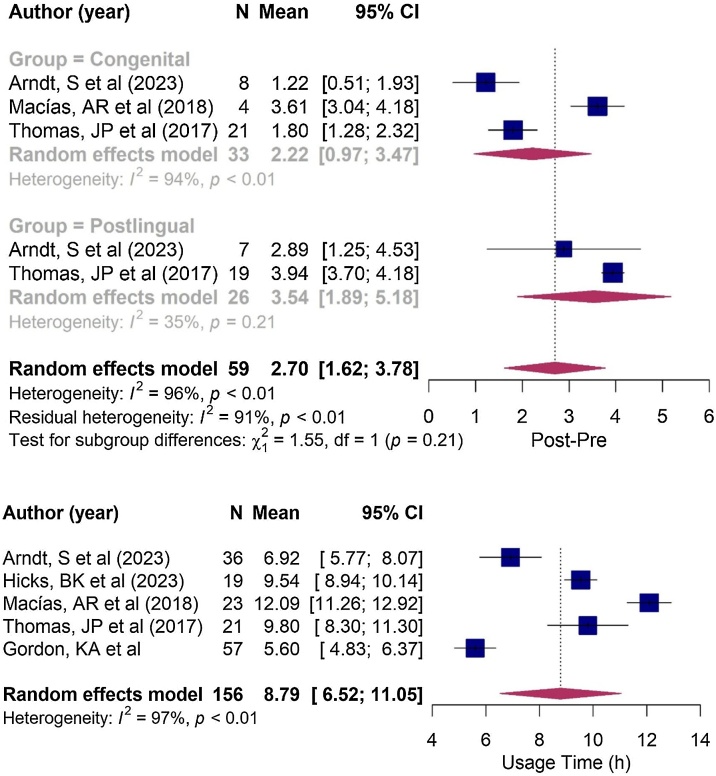
Forest plot illustrating changes in quality of life, as measured by the increase in the mean scores obtained from the SSQ questionnaire after Cochlear Implantation (CI), in relation to the time of onset of hearing loss, as reported by parents. Note that the diamonds intersect the line of similarity, confirming no significant difference between the groups (*p* = 0.21).

The responses from children indicated that CI use increased the QoL scale by an average of 1.51-points (*p*-value < 0.001, 95% CI: 0.84; 2.18), while parental responses showed an average increase of 2.7-points (*p*-value < 0.001, 95% CI: 1.62–3.78; see Fig. 2) considering all periods of hearing loss onset.

**Fig. 3:**
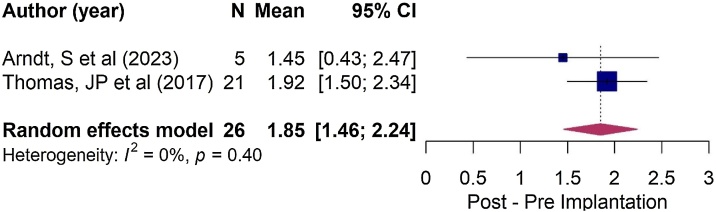
Forest plot illustrating changes in quality of life, as measured by the SSQ questionnaire, after Cochlear implantation (CI) in congenital hearing loss group, as reported by children.

Patients with congenital SSD who underwent CI showed a statistically significant improvement in QoL, as reported by both children and parents. Children reported an average increase of 1.85-points on the SSQ scale (95% CI: 1.46–2.24; see Fig. 3), while parents reported an average increase of 2.22-points on the SSQ scale (95% CI: 0.97–3.47; see Fig. 2).

When assessing patients with post-lingual deafness, caregivers reported a statistically significant improvement in QoL, with a mean increase of 3.54-points on the SSQ scale (95% CI: 1.89–5.18; see Fig. 2).

**Fig. 4:**
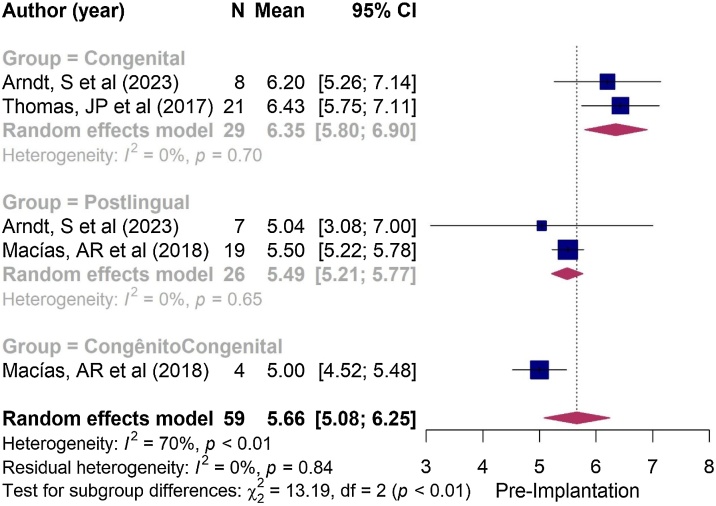
Forest plot illustrating the mean responses obtained from the SSQ questionnaire before Cochlear Implantation (CI) in congenital and post-lingual groups, as reported by parents. Note that the diamonds intersect the line of similarity, confirming no significant difference between the groups (*p* = 0.55).

With regard to the time of onset of hearing loss, no significant difference in QoL was observed between the congenital and post-lingual groups in the parental assessments, both in the pre-implantation period (*p* = 0.550; see Fig. 4) and the postoperative period (*p* = 0.210; see Fig. 2).

**Fig. 5:**
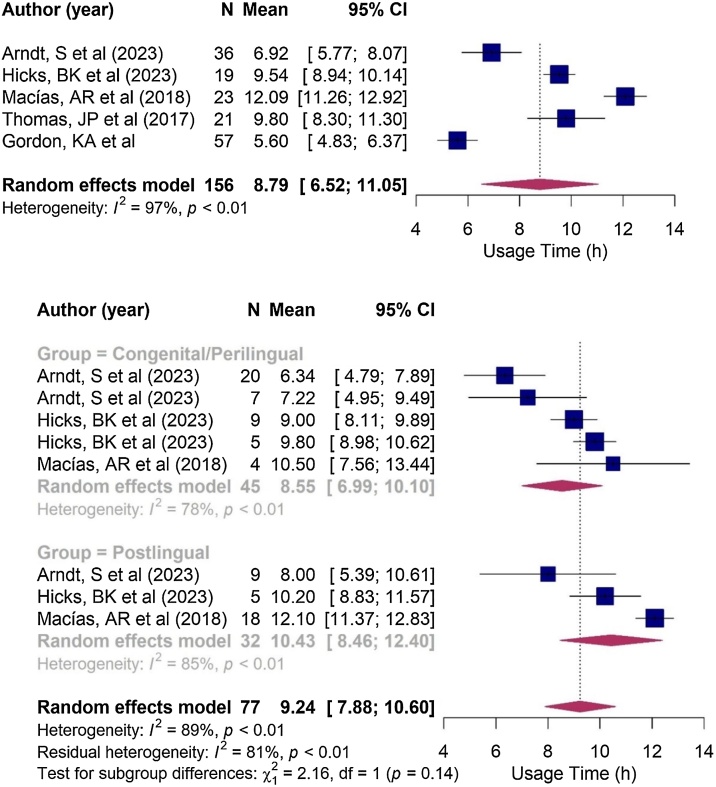
Forest plot illustrating Cochlear Implantation (CI) usage time, in hours per day, not separated by group.

**Fig. 6:**
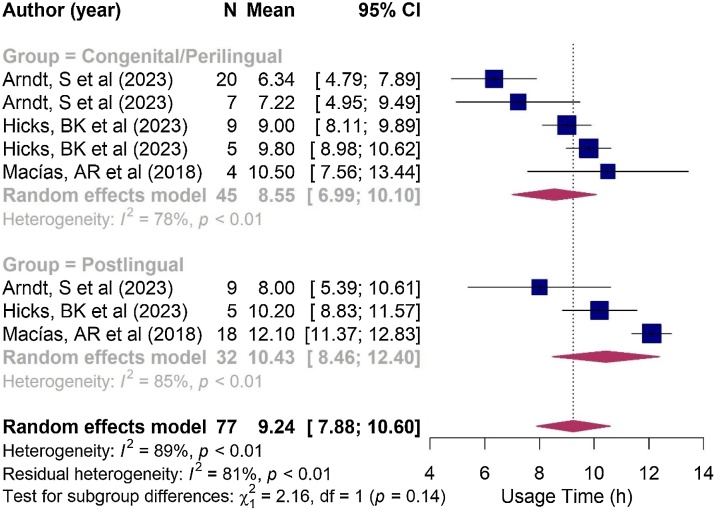
Forest plot comparing the time of use of children in the congenital/perilingual and post-lingual groups. In the Arndt et al.^12^ and Hicks et al.^50^ studies, the first occurrence corresponds to individuals with congenital hearing loss and the second to perilinguals.

The estimated effective usage time of the cochlear implant averaged 8.8 h per day, with a range of 6.52–11.1 h per day (Fig. 5). In the subgroup analysis, the congenital hearing loss group demonstrated a mean usage time of 8.55 h/day (6.99–10.1 h/day), while the post-lingual group reported a mean usage time of 10.43 h/day (8.46–12.4 h/day). However, no significant difference in implant usage time was observed between children with congenital and post-lingual hearing loss (*p* = 0.140; see Fig. 6).

## Discussion

CI for SSD in children has been increasingly accepted as a therapeutic option. However, the subjective aspects associated with implantation in this population remain relatively underexplored. According to the authors, this systematic review with meta-analysis represents the first comprehensive study focused exclusively on this topic, encompassing the largest number of patients evaluated to date. It updates and deepens the findings previously reviewed by Benchetrit et al.^48^ and Nicolas et al.,^68^ which primarily addressed audiological outcomes and different populations, respectively.

The perspectives of children and caregivers were analyzed separately in this study. Three studies directly assessed the perspectives of children. In Arndt et al.,^12^ a comparison of pre- and postoperative periods in children with congenital/perilingual versus post-lingual hearing loss showed favorable absolute scores for the implant in both groups; however, statistical analysis revealed significant improvement only in the post-lingual cohort. Gordon et al.^51^ compared the responses of children with congenital/perilingual hearing loss (referred to as ‘early onset’) to those with post-lingual hearing loss (referred to as ‘late onset’) following surgery, observing improvements in both groups without a statistically significant difference between them. In Thomas et al.,^46^ which focused exclusively on patients with congenital deafness, the SSQ questionnaire for children in the “with” and “without” CI conditions showed improvement with device use. However, statistical significance was not achieved due to the small sample size.

Parental impressions were reviewed in 6 studies.^12^, ^46^, ^49^, ^50^, ^51^, ^69^ All studies reported a significant improvement in QoL with CI. In Thomas et al.,^46^ 84.2% of parents expressed willingness to choose implantation surgery again, and in Hicks et al.,^50^ benefits were maintained or increased even after 24 months post-activation. Therefore, despite the differences in study designs, there is evidence of a positive response to CI in terms of QoL, both from self-assessment and the parental perspectives, findings supported by the meta-analysis.

When comparing parental responses with those of the children, it was observed that parents generally expressed stronger support for the CI. Arndt et al.^12^ attributed the differences to higher parental expectations regarding the outcomes of rehabilitation. In this study, although there was a greater increase in absolute SSQ scores in paternal responses, it is important to note that direct statistical comparisons between parental and children's responses may introduce bias due to differences in questionnaire content and the unequal number of questions across different versions of the SSQ questionnaire.^62^ Nevertheless, the SSQ questionnaire is considered appropriate for evaluation as it assesses binaural hearing functions such as interaural asymmetry and adaptation to unilateral versus bilateral CI.^62^

When evaluating results by time of onset of hearing loss, Arndt et al.^12^ found an improvement in QoL irrespective of the onset or duration of hearing deprivation. Similarly, Marcías et al.^69^ and Gordon et al.^51^ reported a homogeneous improvement in both congenital and post-lingual cases. However, the authors are noted that in post-lingual cases with prolonged deprivation, spatial hearing improvements may be less pronounced, suggesting a maintained of aural preference and, therefore, incomplete restoration of binaural hearing.

According to Zeitler et al.,^49^ the QoL results in the group implanted before or after 7 years of age and with more or less than 7 years of hearing loss are homogeneous, with statistically significant gains in all the groups. Finally, Thomas et al.^46^ found similar results in patients implanted before or after 6-years of age for congenital SSD. Although previous studies^48^ have suggested that patients with congenital hearing loss have worse outcomes than those with post-lingual deafness, in the present analysis revealed improvement in QoL in both groups after CI, with no statistically significant differences between them. Similar responses were observed in the congenital and post-lingual groups also in the preoperative period, suggesting that hearing loss causes comparable impairments in both groups. Therefore, the time of onset and duration of hearing loss should not be absolute contraindications to CI in this population.

Given the current understanding of the optimal neurodevelopmental window, we support the recommendations of Arndt et al.,^12^ which advocate for considering CI as early as possible, aiming for implantation by age 3 in patients with congenital deafness. Additionally, the same author^12^ suggests that for post-lingual patients, there appears to be no strict age limit or timeframe for implantation, provided that expectations regarding the recovery of spatial hearing are appropriately adjusted, with evidence supporting gains up to 7 years of hearing deprivation.

The effective usage time of the device has been evaluated in five studies.^12^, ^46^, ^50^, ^51^^,^^69^ Ardnt et al.^12^ reported 83% regular users over a mean follow-up of 4.75 years. In this study, the patients who became non-users were congenitally deaf and implanted after the age of 3. Thomas et al.^46^ found that 80% of patients used the device daily two years after implantation. Among non-users, there was a correlation between increased educational level and age with a reduction in perceived subjective benefit and significant stigmatization, which led them to stop or limit device use, even after years of effective use. In this review, positive outcomes were observed regarding the implant usability in both congenital and post-lingual populations, the measurement methodologies were often not clearly described.

### Limitations

The small number of studies identified in the review limits the generalizability of the results obtained. This gap in the literature can be explained by the fact that this is a topic of recent interest, involving invasive procedures in a particularly vulnerable population, as well as the expansion of the indications for cochlear implants. Given the sensitivity of the issue and the need for studies on the topic, the contribution of this review is considered relevant, both in terms of understanding the topic and as a stimulus for further studies.

The included studies applied 7 different QoL assessment tools, making it difficult to analyze the data objectively. The SSQ was the most commonly used instrument. To maintain the reliability of the data and reduce bias, it was considered the standard instrument for comparison between the studies. This questionnaire has been adapted and validated for use in both parent and child groups, providing age-appropriate assessments across different age groups. In addition, the scale analyses subjective aspects of spatial hearing, which is particularly important in the reality of SSD. On the other hand, the assessment of other important aspects of QoL, such as impact on interpersonal relationships, emotional and academic issues, are not assessed by the questionnaire.

Statistical analysis of QoL using questionnaires directly applied to patients with post-lingual deafness was not feasible due to limited data availability. Only one study, conducted by Arndt et al.,^12^ explored this aspect.

The absence of standardized definitions for congenital, perilingual, and post-lingual hearing loss in the literature hindered direct comparisons between studies. In this review, available individual data were analyzed to standardize classification criteria. Additionally, detailed analysis of specific pathologies associated with questionnaire responses was not feasible due to lack of data.

To better understand the issue, future studies should consider developing a more comprehensive QoL assessment tool in the pediatric SSD population. Clear definitions of time to installation of deafness may help to ensure directly comparable data and increase the validity of the results. Investigation of CI outcomes in specific pathologies is imprescindible to determine which population may benefit with the CI. Larger cohorts and longer follow-up studies are necessary to comprehensively assess long-term adherence. Special attention is warranted for the adolescent population, where social issues pose a significant risk of discontinuing device use.

In order to enhance comprehension, the issue and facilitate the execution of new meta-analyses comprising a more substantial number of studies and definitive conclusions, the authors of this review strongly encourage the development of new study groups and follow-up publications of cohorts already already under observation. Future studies should consider developing a more comprehensive QoL assessment tool for the pediatric SSD population, covering subjective aspects in addition to those directly related to hearing. Clear definitions of the time to onset of deafness, as well as providing the ages of onset of hearing loss and implantation, could help to ensure directly comparable data and increase the validity of the results. Research into the outcomes of CI in specific pathologies, both pre- and post-lingually deaf, is essential to determine which population may benefit from CI. Larger cohorts and longer follow-up studies are needed to comprehensively assess long-term adherence and to delimit populations and reasons for CI discontinuation. Special attention is needed for the adolescent population, where social issues represent a significant risk of discontinuing use of the device.

## Conclusion

This review demonstrated a significant improvement in Quality of Life (QoL) following cochlear implantation in children with Single-Sided Deafness (SSD), as reported by both parents and the children themselves. Notable adherence to treatment was also observed. Patients with congenital and post-lingual hearing loss exhibited similar improvements in QoL and in the duration of effective device usage. Larger cohorts and longer follow-up studies are necessary to confirm these findings and to more thoroughly evaluate factors that affect long-term discontinuation of device use, particularly in teenagers.

## Funding

No financing funds were used.

## Declaration of competing interest

The authors declare no conflicts of interest.
