# Supplementary material for: Quality of life in children with unilateral hearing loss undergoing cochlear implantation: A systematic review and meta-analysis
Source: Braz J Otorhinolaryngol. 2025 May 14;91(5):101628. doi: 10.1016/j.bjorl.2025.101628 (PMC12144431; doi:10.1016/j.bjorl.2025.101628)
Supplement: Supplementary file 1 [file mmc1.docx]

BJORL-D-24-00350_Supplementary Material

**Supplemental Data**

**Supplement 1** Detailed search strategies for platform.

| **Source** | **Strategy** | **Number of articles 11/08/2023** | **Update 15/12/2023** |
| --- | --- | --- | --- |
| PUBMED | ((((((Infant OR Infants) OR ("Child, Preschool" OR "Preschool Child" OR "Children, Preschool" OR "Preschool Children")) OR (Child OR Children)) OR (Adolescent OR Adolescents OR Adolescence OR Teens OR Teen OR Teenagers OR Teenager OR Youth OR Youths OR "Adolescents, Female" OR "Adolescent, Female" OR "Female Adolescent" OR "Female Adolescents" OR "Adolescents, Male" OR "Adolescent, Male" OR "Male Adolescent" OR "Male Adolescents")) AND (((((Hearing Loss, Unilateral[MeSH Terms]) OR ("Hearing Loss, Unilateral"[Title/Abstract] OR "Unilateral Hearing Loss"[Title/Abstract] OR "Deafness, Unilateral"[Title/Abstract] OR "Unilateral Deafness"[Title/Abstract] OR "Deafness Unilateral"[Title/Abstract] OR "Unilateral, Deafness"[Title/Abstract])) OR ("single‒sided deafness"[Title/Abstract] OR "Single‒sided deafness (SSD)"[Title/Abstract] OR "single‒sided deafness"[Title/Abstract])) OR ("unilateral sensorineural hearing loss"[Title/Abstract])) OR ((((Deafness[MeSH Terms]) OR (Deafness[Title/Abstract] OR "Hearing Loss, Complete"[Title/Abstract] OR "Complete Hearing Loss"[Title/Abstract] OR "Hearing Loss, Extreme"[Title/Abstract] OR "Extreme Hearing Loss"[Title/Abstract] OR "Prelingual Deafness"[Title/Abstract] OR "Deafness, Prelingual"[Title/Abstract] OR "Deafness, Acquired"[Title/Abstract] OR "Acquired Deafness"[Title/Abstract] OR "Deafness Permanent"[Title/Abstract] OR "Permanent, Deafness"[Title/Abstract] OR "Permanents, Deafness"[Title/Abstract] OR "Hearing Loss Permanent"[Title/Abstract] OR "Permanent, Hearing Loss"[Title/Abstract] OR "Deaf Mutism"[Title/Abstract] OR "Deaf‒Mutism"[Title/Abstract])) OR ((Hearing Loss[MeSH Terms]) OR ("Hearing Loss"[Title/Abstract] OR "Loss, Hearing"[Title/Abstract] OR Hypoacusis[Title/Abstract] OR Hypoacuses[Title/Abstract] OR "Hearing Impairment"[Title/Abstract]))) AND ((monoaural[Title/Abstract] OR monaural[Title/Abstract]) OR ("monaural hearing"[Title/Abstract]))))) AND ((Cochlear Implantation[MeSH Terms]) OR ("Cochlear Implantation"[Title/Abstract] OR "Cochlear Implantations"[Title/Abstract] OR "Implantation, Cochlear"[Title/Abstract] OR "Implantations, Cochlear"[Title/Abstract] OR "Cochlear Prosthesis Implantation"[Title/Abstract] OR "Cochlear Prosthesis Implantations"[Title/Abstract] OR "Implantation, Cochlear Prosthesis"[Title/Abstract] OR "Implantations, Cochlear Prosthesis"[Title/Abstract] OR "Prosthesis Implantation, Cochlear"[Title/Abstract] OR "Prosthesis Implantations, Cochlear"[Title/Abstract]))) AND (((Quality of Life[MeSH Terms]) OR ("Quality of Life"[Title/Abstract] OR "Life Quality"[Title/Abstract] OR "Health‒Related Quality Of Life"[Title/Abstract] OR "Health Related Quality Of Life"[Title/Abstract] OR HRQOL[Title/Abstract])) OR ("Psychosocial Impact"[Title/Abstract])) | **22** | **25** |
| PUBMED PMC | ((((((Infant OR Infants) OR ("Child, Preschool" OR "Preschool Child" OR "Children, Preschool" OR "Preschool Children")) OR (Child OR Children)) OR (Adolescent OR Adolescents OR Adolescence OR Teens OR Teen OR Teenagers OR Teenager OR Youth OR Youths OR "Adolescents, Female" OR "Adolescent, Female" OR "Female Adolescent" OR "Female Adolescents" OR "Adolescents, Male" OR "Adolescent, Male" OR "Male Adolescent" OR "Male Adolescents")) AND (((((Hearing Loss, Unilateral[MeSH Terms]) OR ("Hearing Loss, Unilateral"[Title/Abstract] OR "Unilateral Hearing Loss"[Title/Abstract] OR "Deafness, Unilateral"[Title/Abstract] OR "Unilateral Deafness"[Title/Abstract] OR "Deafness Unilateral"[Title/Abstract] OR "Unilateral, Deafness"[Title/Abstract])) OR ("single‒sided deafness"[Title/Abstract] OR "Single‒sided deafness (SSD)"[Title/Abstract] OR "single‒sided deafness"[Title/Abstract])) OR ("unilateral sensorineural hearing loss"[Title/Abstract])) OR ((((Deafness[MeSH Terms]) OR (Deafness[Title/Abstract] OR "Hearing Loss, Complete"[Title/Abstract] OR "Complete Hearing Loss"[Title/Abstract] OR "Hearing Loss, Extreme"[Title/Abstract] OR "Extreme Hearing Loss"[Title/Abstract] OR "Prelingual Deafness"[Title/Abstract] OR "Deafness, Prelingual"[Title/Abstract] OR "Deafness, Acquired"[Title/Abstract] OR "Acquired Deafness"[Title/Abstract] OR "Deafness Permanent"[Title/Abstract] OR "Permanent, Deafness"[Title/Abstract] OR "Permanents, Deafness"[Title/Abstract] OR "Hearing Loss Permanent"[Title/Abstract] OR "Permanent, Hearing Loss"[Title/Abstract] OR "Deaf Mutism"[Title/Abstract] OR "Deaf‒Mutism"[Title/Abstract])) OR ((Hearing Loss[MeSH Terms]) OR ("Hearing Loss"[Title/Abstract] OR "Loss, Hearing"[Title/Abstract] OR Hypoacusis[Title/Abstract] OR Hypoacuses[Title/Abstract] OR "Hearing Impairment"[Title/Abstract]))) AND ((monoaural[Title/Abstract] OR monaural[Title/Abstract]) OR ("monaural hearing"[Title/Abstract]))))) AND ((Cochlear Implantation[MeSH Terms]) OR ("Cochlear Implantation"[Title/Abstract] OR "Cochlear Implantations"[Title/Abstract] OR "Implantation, Cochlear"[Title/Abstract] OR "Implantations, Cochlear"[Title/Abstract] OR "Cochlear Prosthesis Implantation"[Title/Abstract] OR "Cochlear Prosthesis Implantations"[Title/Abstract] OR "Implantation, Cochlear Prosthesis"[Title/Abstract] OR "Implantations, Cochlear Prosthesis"[Title/Abstract] OR "Prosthesis Implantation, Cochlear"[Title/Abstract] OR "Prosthesis Implantations, Cochlear"[Title/Abstract]))) AND (((Quality of Life[MeSH Terms]) OR ("Quality of Life"[Title/Abstract] OR "Life Quality"[Title/Abstract] OR "Health‒Related Quality Of Life"[Title/Abstract] OR "Health Related Quality Of Life"[Title/Abstract] OR HRQOL[Title/Abstract])) OR ("Psychosocial Impact"[Title/Abstract])) | **10** | **10** |
| BVS – BIREME MEDLINE (21) | ((infant OR infants) OR ("Child, Preschool" OR "Preschool Child" OR "Children, Preschool" OR "Preschool Children") OR (child OR children) OR (adolescent OR adolescents OR adolescence OR teens OR teen OR teenagers OR teenager OR youth OR youths OR "Adolescents, Female" OR "Adolescent, Female" OR "Female Adolescent" OR "Female Adolescents" OR "Adolescents, Male" OR "Adolescent, Male" OR "Male Adolescent" OR "Male Adolescents")) AND ((("Hearing Loss, Unilateral" OR "Unilateral Hearing Loss" OR "Deafness, Unilateral" OR "Unilateral Deafness" OR "Deafness Unilateral" OR "Unilateral, Deafness") OR ("single‒sided deafness" OR "Single‒sided deafness (SSD)" OR "single‒sided deafness") OR ("unilateral sensorineural hearing loss")) OR (((Deafness OR "Hearing Loss, Complete" OR "Complete Hearing Loss" OR "Hearing Loss, Extreme" OR "Extreme Hearing Loss" OR "Prelingual Deafness" OR "Deafness, Prelingual" OR "Deafness, Acquired" OR "Acquired Deafness" OR "Deafness Permanent" OR "Permanent, Deafness" OR "Permanents, Deafness" OR "Hearing Loss Permanent" OR "Permanent, Hearing Loss" OR "Deaf Mutism" OR "Deaf‒Mutism") OR ("Hearing Loss" OR "Loss, Hearing" OR hypoacusis OR hypoacuses OR "Hearing Impairment")) AND ((monoaural OR monaural) OR ("monaural hearing" )))) AND (("Cochlear Implantation" OR "Cochlear Implantations" OR "Implantation, Cochlear" OR "Implantations, Cochlear" OR "Cochlear Prosthesis Implantation" OR "Cochlear Prosthesis Implantations" OR "Implantation, Cochlear Prosthesis" OR "Implantations, Cochlear Prosthesis" OR "Prosthesis Implantation, Cochlear" OR "Prosthesis Implantations, Cochlear")) AND (("Quality of Life" OR "Life Quality" OR "Health‒Related Quality Of Life" OR "Health Related Quality Of Life" OR hrqol) OR ("Psychosocial Impact")) | **21** | **24** |
| EBSCOHOST | (Infant OR Infants) OR ("Child, Preschool" OR "Preschool Child" OR "Children, Preschool" OR "Preschool Children") OR (Child OR Children) OR (Teenager OR Youth OR Youths OR "Adolescents, Female" OR "Adolescent, Female" OR "Female Adolescent" OR "Female Adolescents" OR "Adolescents, Male" OR "Adolescent, Male" OR "Male Adolescent" OR "Male Adolescents") AND ("Hearing Loss, Unilateral" OR "Unilateral Hearing Loss" OR "Deafness, Unilateral" OR "Unilateral Deafness" OR "Deafness Unilateral" OR "Unilateral, Deafness") OR ("single‒sided deafness" OR "Single‒sided deafness (SSD)" OR "single‒sided deafness") OR "unilateral sensorineural hearing loss" OR (Deafness OR "Hearing Loss, Complete" OR "Complete Hearing Loss" OR "Hearing Loss, Extreme" OR "Extreme Hearing Loss" OR "Prelingual Deafness" OR "Deafness, Prelingual" OR "Deafness, Acquired" OR "Acquired Deafness" OR "Deafness Permanent" OR "Permanent, Deafness" OR "Permanents, Deafness" OR "Hearing Loss Permanent" OR "Permanent, Hearing Loss" OR "Deaf Mutism" OR "Deaf‒Mutism") OR ("Hearing Loss" OR "Loss, Hearing" OR Hypoacusis OR Hypoacuses OR "Hearing Impairment") AND (monoaural OR monaural) OR "monaural hearing" AND "Cochlear Implantation" OR "Cochlear Implantations" OR "Implantation, Cochlear" OR "Implantations, Cochlear" OR "Cochlear Prosthesis Implantation" OR "Cochlear Prosthesis Implantations" OR "Implantation, Cochlear Prosthesis" OR "Implantations, Cochlear Prosthesis" OR "Prosthesis Implantation, Cochlear" OR "Prosthesis Implantations, Cochlear" AND ("Quality of Life" OR "Life Quality" OR "Health‒Related Quality Of Life" OR "Health Related Quality Of Life" OR HRQOL) OR "Psychosocial Impact" | **30** | **30** |
| SCOPUS | (((TITLE‒ABS‒KEY ( infant OR infants) OR ALL ("Child, Preschool" OR "Preschool Child" OR "Children, Preschool" OR "Preschool Children") OR ALL (child OR children) OR TITLE‒ABS‒KEY (adolescent OR adolescents OR adolescence OR teens OR teen OR teenagers OR teenager OR youth OR youths OR "Adolescents, Female" OR "Adolescent, Female" OR "Female Adolescent" OR "Female Adolescents" OR "Adolescents, Male" OR "Adolescent, Male" OR "Male Adolescent" OR "Male Adolescents"))) AND (((TITLE‒ABS‒KEY ("Hearing Loss, Unilateral" OR "Unilateral Hearing Loss" OR "Deafness, Unilateral" OR "Unilateral Deafness" OR "Deafness Unilateral" OR "Unilateral, Deafness") OR TITLE‒ABS‒KEY ("single‒sided deafness" OR "Single‒sided deafness (SSD)" OR "single‒sided deafness") OR TITLE‒ABS‒KEY ("unilateral sensorineural hearing loss"))) OR (((TITLE‒ABS‒KEY (deafness OR "Hearing Loss, Complete" OR "Complete Hearing Loss" OR "Hearing Loss, Extreme" OR "Extreme Hearing Loss" OR "Prelingual Deafness" OR "Deafness, Prelingual" OR "Deafness, Acquired" OR "Acquired Deafness" OR "Deafness Permanent" OR "Permanent, Deafness" OR "Permanents, Deafness" OR "Hearing Loss Permanent" OR "Permanent, Hearing Loss" OR "Deaf Mutism" OR "Deaf‒Mutism") OR TITLE‒ABS‒KEY ("Hearing Loss" OR "Loss, Hearing" OR hypoacusis OR hypoacuses OR "Hearing Impairment"))) AND ((TITLE‒ABS‒KEY (monoaural OR monaural) OR TITLE‒ABS‒KEY ("monaural hearing")))))) AND (TITLE‒ABS‒KEY ("Cochlear Implantation" OR "Cochlear Implantations" OR "Implantation, Cochlear" OR "Implantations, Cochlear" OR "Cochlear Prosthesis Implantation" OR "Cochlear Prosthesis Implantations" OR "Implantation, Cochlear Prosthesis" OR "Implantations, Cochlear Prosthesis" OR "Prosthesis Implantation, Cochlear" OR "Prosthesis Implantations, Cochlear")) AND ((TITLE‒ABS‒KEY ("Quality of Life" OR "Life Quality" OR "Health‒Related Quality Of Life" OR "Health Related Quality Of Life" OR hrqol) OR TITLE‒ABS‒KEY ("Psychosocial Impact"))) | **81** | **89** |
| Web of Science | Infant OR Infants (Topic) or "Child, Preschool" OR "Preschool Child" OR "Children, Preschool" OR "Preschool Children" (Topic) or Child OR Children (Topic) or Adolescent OR Adolescents OR Adolescence OR Teens OR Teen OR Teenagers OR Teenager OR Youth OR Youths OR "Adolescents, Female" OR "Adolescent, Female" OR "Female Adolescent" OR "Female Adolescents" OR "Adolescents, Male" OR "Adolescent, Male" OR "Male Adolescent" OR "Male Adolescents" (Topic) and Preprint Citation Index (Exclude – Database) AND "Hearing Loss, Unilateral" OR "Unilateral Hearing Loss" OR "Deafness, Unilateral" OR "Unilateral Deafness" OR "Deafness Unilateral" OR "Unilateral, Deafness" (Topic) or "single‒sided deafness" OR "Single‒sided deafness (SSD)" OR "single‒sided deafness" (Topic) or "unilateral sensorineural hearing loss" (Topic) and Preprint Citation Index (Exclude – Database) OR Deafness OR "Hearing Loss, Complete" OR "Complete Hearing Loss" OR "Hearing Loss, Extreme" OR "Extreme Hearing Loss" OR "Prelingual Deafness" OR "Deafness, Prelingual" OR "Deafness, Acquired" OR "Acquired Deafness" OR "Deafness Permanent" OR "Permanent, Deafness" OR "Permanents, Deafness" OR "Hearing Loss Permanent" OR "Permanent, Hearing Loss" OR "Deaf Mutism" OR "Deaf‒Mutism" (Topic) or "Hearing Loss" OR "Loss, Hearing" OR Hypoacusis OR Hypoacuses OR "Hearing Impairment" (Topic) and Preprint Citation Index (Exclude – Database) AND monoaural OR monaural (Topic) or "monaural hearing" (Topic) and Preprint Citation Index (Exclude – Database) AND "Cochlear Implantation" OR "Cochlear Implantations" OR "Implantation, Cochlear" OR "Implantations, Cochlear" OR "Cochlear Prosthesis Implantation" OR "Cochlear Prosthesis Implantations" OR "Implantation, Cochlear Prosthesis" OR "Implantations, Cochlear Prosthesis" OR "Prosthesis Implantation, Cochlear" OR "Prosthesis Implantations, Cochlear" (Topic) and Preprint Citation Index (Exclude – Database) AND "Quality of Life" OR "Life Quality" OR "Health‒Related Quality Of Life" OR "Health Related Quality Of Life" OR HRQOL (Topic) or "Psychosocial Impact" (Topic) and Preprint Citation Index (Exclude – Database) | **65** | **69** |
| EMBASE | (infant OR infants OR 'child, preschool' OR 'preschool child' OR 'children, preschool' OR 'preschool children' OR child OR children OR adolescent OR adolescents OR adolescence OR teens OR teen OR teenagers OR teenager OR youth OR youths OR 'adolescents, female' OR 'adolescent, female' OR 'female adolescent' OR 'female adolescents' OR 'adolescents, male' OR 'adolescent, male' OR 'male adolescent' OR 'male adolescents') AND ('unilateral hearing loss'/syn OR ('single‒sided deafness':ti,ab,kw AND ssd:ti,ab,kw) OR 'single‒sided deafness':ti,ab,kw OR 'unilateral sensorineural hearing loss':ti,ab,kw OR ('hearing impairment'/syn AND (monoaural:ti,ab,kw OR monaural:ti,ab,kw OR 'monaural hearing'/syn))) AND 'cochlear implantation'/syn AND ('quality of life'/syn OR 'psychosocial impact':ti,ab,kw) | **30** | **34** |
| Cochrane Library | (Infant OR Infants):ti,ab,kw OR ("Child, Preschool" OR "Preschool Child" OR "Children, Preschool" OR "Preschool Children"):ti,ab,kw OR (Child OR Children):ti,ab,kw OR (Adolescent OR Adolescents OR Adolescence OR Teens OR Teen OR Teenagers OR Teenager OR Youth OR Youths OR "Adolescents, Female" OR "Adolescent, Female" OR "Female Adolescent" OR "Female Adolescents" OR "Adolescents, Male" OR "Adolescent, Male" OR "Male Adolescent" OR "Male Adolescents"):ti,ab,kw AND MeSH descriptor: [Hearing Loss, Unilateral] explode all trees OR ("Hearing Loss, Unilateral" OR "Unilateral Hearing Loss" OR "Deafness, Unilateral" OR "Unilateral Deafness" OR "Deafness Unilateral" OR "Unilateral, Deafness"):ti,ab,kw OR ("single sided deafness" OR "Single sided deafness (SSD)" OR "single sided deafness"):ti,ab,kw OR ("unilateral sensorineural hearing loss"):ti,ab,kw AND MeSH descriptor: [Deafness] explode all trees OR (Deafness OR "Hearing Loss, Complete" OR "Complete Hearing Loss" OR "Hearing Loss, Extreme" OR "Extreme Hearing Loss" OR "Prelingual Deafness" OR "Deafness, Prelingual" OR "Deafness, Acquired" OR "Acquired Deafness" OR "Deafness Permanent" OR "Permanent, Deafness" OR "Permanents, Deafness" OR "Hearing Loss Permanent" OR "Permanent, Hearing Loss" OR "Deaf Mutism" OR "Deaf‒Mutism"):ti,ab,kw OR ("Hearing Loss" OR "Loss, Hearing" OR Hypoacusis OR Hypoacuses OR "Hearing Impairment"):ti,ab,kw OR MeSH descriptor: [Hearing Loss] explode all trees AND (monoaural OR monaural):ti,ab,kw OR ("monaural hearing"):ti,ab,kw AND MeSH descriptor: [Cochlear Implantation] explode all trees OR ("Cochlear Implantation" OR "Cochlear Implantations" OR "Implantation, Cochlear" OR "Implantations, Cochlear" OR "Cochlear Prosthesis Implantation" OR "Cochlear Prosthesis Implantations" OR "Implantation, Cochlear Prosthesis" OR "Implantations, Cochlear Prosthesis" OR "Prosthesis Implantation, Cochlear" OR "Prosthesis Implantations, Cochlear"):ti,ab,kw AND MeSH descriptor: [Quality of Life] explode all trees OR ("Quality of Life" OR "Life Quality" OR "Health‒Related Quality Of Life" OR "Health Related Quality Of Life" OR HRQOL):ti,ab,kw OR ("Psychosocial Impact"):ti,ab,kw | 01 | **01** |
| PROQUEST | (Infant OR Infants) OR ("Child, Preschool" OR "Preschool Child" OR "Children, Preschool" OR "Preschool Children") OR (Child OR Children) OR (Adolescent OR Adolescents OR Adolescence OR Teens OR Teen OR Teenagers OR Teenager OR Youth OR Youths OR "Adolescents, Female" OR "Adolescent, Female" OR "Female Adolescent" OR "Female Adolescents" OR "Adolescents, Male" OR "Adolescent, Male" OR "Male Adolescent" OR "Male Adolescents") AND abstract("Hearing Loss, Unilateral" OR "Unilateral Hearing Loss" OR "Deafness, Unilateral" OR "Unilateral Deafness" OR "Deafness Unilateral" OR "Unilateral, Deafness") OR title("Hearing Loss, Unilateral" OR "Unilateral Hearing Loss" OR "Deafness, Unilateral" OR "Unilateral Deafness" OR "Deafness Unilateral" OR "Unilateral, Deafness") OR abstract("single‒sided deafness" OR "Single‒sided deafness (SSD)" OR "single‒sided deafness") OR title("single‒sided deafness" OR "Single‒sided deafness (SSD)" OR "single‒sided deafness") OR abstract("unilateral sensorineural hearing loss") OR title("unilateral sensorineural hearing loss") OR abstract( Deafness OR "Hearing Loss, Complete" OR "Complete Hearing Loss" OR "Hearing Loss, Extreme" OR "Extreme Hearing Loss" OR "Prelingual Deafness" OR "Deafness, Prelingual" OR "Deafness, Acquired" OR "Acquired Deafness" OR "Deafness Permanent" OR "Permanent, Deafness" OR "Permanents, Deafness" OR "Hearing Loss Permanent" OR "Permanent, Hearing Loss" OR "Deaf Mutism" OR "Deaf‒Mutism") OR title( Deafness OR "Hearing Loss, Complete" OR "Complete Hearing Loss" OR "Hearing Loss, Extreme" OR "Extreme Hearing Loss" OR "Prelingual Deafness" OR "Deafness, Prelingual" OR "Deafness, Acquired" OR "Acquired Deafness" OR "Deafness Permanent" OR "Permanent, Deafness" OR "Permanents, Deafness" OR "Hearing Loss Permanent" OR "Permanent, Hearing Loss" OR "Deaf Mutism" OR "Deaf‒Mutism") OR abstract( "Hearing Loss" OR "Loss, Hearing" OR Hypoacusis OR Hypoacuses OR "Hearing Impairment") OR title( "Hearing Loss" OR "Loss, Hearing" OR Hypoacusis OR Hypoacuses OR "Hearing Impairment") AND abstract( monoaural OR monaural) OR title( monoaural OR monaural) OR abstract( "monaural hearing" ) OR title( "monaural hearing" ) AND abstract("Cochlear Implantation" OR "Cochlear Implantations" OR "Implantation, Cochlear" OR "Implantations, Cochlear" OR "Cochlear Prosthesis Implantation" OR "Cochlear Prosthesis Implantations" OR "Implantation, Cochlear Prosthesis" OR "Implantations, Cochlear Prosthesis" OR "Prosthesis Implantation, Cochlear" OR "Prosthesis Implantations, Cochlear") OR title("Cochlear Implantation" OR "Cochlear Implantations" OR "Implantation, Cochlear" OR "Implantations, Cochlear" OR "Cochlear Prosthesis Implantation" OR "Cochlear Prosthesis Implantations" OR "Implantation, Cochlear Prosthesis" OR "Implantations, Cochlear Prosthesis" OR "Prosthesis Implantation, Cochlear" OR "Prosthesis Implantations, Cochlear") AND abstract( "Quality of Life" OR "Life Quality" OR "Health‒Related Quality Of Life" OR "Health Related Quality Of Life" OR HRQOL) OR title( "Quality of Life" OR "Life Quality" OR "Health‒Related Quality Of Life" OR "Health Related Quality Of Life" OR HRQOL) OR abstract("Psychosocial Impact") OR title("Psychosocial Impact") | **10** | **10** |
| **Total** |  | 270 | **292** |
| Total duplicate references | 145 articles excluded for duplicates on Rayyan | **145** | **149** |
| Total after deleting duplicates in endnote |  | 125 | **143** |
| Total Duplicate references in endnote web | 10 articles excluded for duplicates on EndNote Web |  | **10** |
| Total after deleting duplicates in Rayyan |  |  | **133** |

**Supplement 2** Reasons for exclusions after reading the full text.

| **Reason for exclusion** | **Excluded article** | |
| --- | --- | --- |
| Wrong population (n = 12) | Tolisano, 2023 | Galvin, 2019 |
|  | Ullah, 2023 | Marx, 2019 |
|  | Plath, 2022 | Távora-Vieira, 2019 |
|  | Tan, 2022 | Dorbeau, 2018 |
|  | Seebacher, 2021 | Mertens, 2015 |
|  | Häussler, 2020 | Ramos Macías, 2015 |
| Overlapping Cohorts (n = 3) | Beck, 2017 | |
|  | Brown, 2022 | |
|  | Arndt, 2022 | |
| Non‒SSD hearing loss (n = 3) | Piromchai, 2021 | |
|  | Schaefer, 2019 | |
|  | Sach, 2007 | |
| Not Evaluated Outcome of Interest (n = 3) | Ehrmann-Mueller, 2020 | |
|  | van Wieringen, 2019 | |
|  | Rahne, 2016 | |
| Inappropriate Method of Intervention (n = 3) | McSweeny, 2021 | |
|  | Overgaard, 2021 | |
|  | Rohlfs, 2017 | |
| Children's Data Not Separated from Adults (n = 2) | Sladen, 2017 | |
|  | Quatre, 2020 | |
| Type of Publication Inadequate (n = 2) | Yu, 2020 | |
|  | Gumus, 2023 | |

**Supplement 3** Newcastle‒Ottawa quality assessment scale for cohort studies, modified for the needs of the review.

| **Selection** |
| --- |
| 1. Representativeness of the exposed cohort |
| a) Truly representative of the average of ***children with severe to profound unilateral hearing loss*** in the community* |
| b) Somewhat representative of ***the*** average of ***children with severe to profound unilateral hearing loss*** in the community* |
| c) Selected group of users |
| d) No description of the cohort derivation |
| 2. Selection of the unexposed cohort (children with unilateral hearing loss without rehabilitation) |
| a) From the same community as the exposed cohort* |
| b) Taken from a different source |
| c) No description of the derivation of the unexposed cohort |
| 3. Exposure verification (***hearing rehabilitation by cochlear implant***) |
| a) Secure registration* |
| b) Structured interview* |
| c) Written self‒report |
| d) No description |
| 4. Demonstration that the outcome of interest was not present at the start of the study (**quality of life**) |
| a) Yes* |
| b) No |
|  |
| **Comparability** |
| 1. Comparability of cohorts based on design or analysis |
| a) Study controls for severe to profound hearing loss* |
| b) Study controls for any additional factors* |
|  |
| **Results** |
| 1. Evaluating the result |
| a) Independent blind assessment* |
| b) Record linkage |
| c) Self‒report |
| d) No description |
| 2. The follow‒up was long enough for the results to occur |
| a) Yes (**more than 3-months**)* |
| b) No |
| 3. Adequate follow-up of cohorts |
| a) complete follow-up ‒ all subjects accounted for* |
| b) Individuals lost to follow‒up with low probability of introducing bias ‒ small number lost ‒ >**80**%* |
| c) Follow‒up rate <**80%** and no description of those lost |
| d) No declaration |

**Supplement 4** Newcastle‒Ottawa quality assessment scale for cohort studies, modified for the needs of the review.

| **Selection** |
| --- |
| 1. Is the case definition adequate? |
| a) Yes, with independent validation* |
| b) Yes, for example, linking records or based on self‒reports |
| c) No description |
| 2. Representativeness of cases |
| a) Consecutive or obviously representative case series* |
| b) Potential for selection bias or undeclared |
| 3. Selecting Controls |
| a) Community controls* |
| b) Hospital controls |
| c) No description |
| 4. Definition of Controls |
| a) No history of illness* |
| b) No source description |
|  |
| **Comparability** |
| 1. Comparability of cases and controls based on the project or analysis |
| a) Study controls for *unilateral severe to profound hearing loss** |
| b) Study controls for any additional factors* |
|  |
| **Exhibition** |
| 1. Exposure check |
| a) Secure registration* |
| b) Structured interview where case/control status is blind* |
| c) Interview not blind to case/control status |
| d) Written self‒report or medical records only |
| e) No description |
| 2. Same calculation method for cases and controls |
| a) Yes* |
| b) No |
| 3. Non‒response rate |
| a) Same rate for both groups* |
| b) No interviewees described |
| c) Different rate and no designation |

**Supplement 5** Quality assessment of studies according to the Newcastle‒Ottawa Scale.

| **Newcastle ‒ Ottawa Criteria** | | **Author, year** | | | | | |
| --- | --- | --- | --- | --- | --- | --- | --- |
|  |  | **Hicks, 2023** | **Zeitler, 2023** | **Arndt, 2023** | **Macías, 2019** | **Thomas, 2017** | **Gordon, 2023** |
| Selection | Representativeness of the exposed cohort | * | * | * | * | * | * |
|  | Selection of unexposed cohort | NA | * | NA | NA | NA | _ |
|  | Exposure check | * | * | * | * | * | * |
|  | Outcome of interest not present at start of exposure | * | * | * | * | * | * |
| Comparability | Comparability of cohorts based on design or analysis | NA | * | NA | NA | NA | * |
|  | Evaluating the result | * | * | * | * | * | * |
| Outcome | Long enough follow-up | * | * | * | * | * | * |
|  | Proper monitoring of cohorts | * | NA | * | * | * | * |
| Total | | 6 | 7 | 6 | 7 | 6 | 7 |

**Supplement 6** Characteristics of the studies evaluated: analysis quantitative.

| **Features** | **Category** | **Arndt (2023)^a^** | **Hicks (2023)** | **Macías (2019)^a^** | **Thomas (2017)^a^** | **Zeitler (2023)** | **Gordon (2023)** | **Mean** | **Minimum** | **Maximum** |
| --- | --- | --- | --- | --- | --- | --- | --- | --- | --- | --- |
| Population | N | 36 | 19 | 23 | 21 | 31 | 57 | 31.2 | 19 | 57 |
|  | Male | 21 | 11 | 11 | 8 | 13 | ‒ | 12.8 | 8 | 21 |
|  | Female | 15 | 8 | 12 | 13 | 17 | ‒ | 13.0 | 8 | 17 |
|  | Missing | 0 | 0 | 0 | 0 | 1 | 0 |  |  |  |
| Time of onset of hearing loss | Congenital | 20 | 9 | 4 | 21 | 12 | 40 | 17.7 | 4 | 40 |
|  | Perilingual (0‒4a) | 7 | 5 | 1 | 0 | 13 | ‒ | 5.2 | 0 | 13 |
|  | Postlingual (>4a) | 9 | 5 | 18 | 0 | 0 | 17 | 8.2 | 0 | 18 |
|  | Missing | 0 | 0 | 0 | 0 | 6 | 0 |  |  |  |
| Age at implantation | <1‒year | 0 | 0 | 1 | 2 | ‒ | ‒ |  |  |  |
|  | 01‒02y | 8 | 0 | 1 | 2 | ‒ | ‒ |  |  |  |
|  | 02‒03y | 2 | 0 | 0 | 1 | ‒ | ‒ |  |  |  |
|  | 03‒04y | 2 | 6 | 2 | 1 | ‒ | ‒ |  |  |  |
|  | 04‒05y | 7 | 3 | 2 | 5 | ‒ | ‒ |  |  |  |
|  | <5-years | 19 | 9 | 6 | 11 | ‒ | ‒ |  |  |  |
|  | >5-years | 17 | 10 | 17 | 10 | 13 | ‒ |  |  |  |
|  | Avarege age (years) | 6.1 | 5.2 | 6.01 | 5.6 | 6.73 | 5.21 | 5.8 | 5.2 | 6.7 |
|  | Missing | 0 | 0 | 0 | 0 | 1 | 0 |  |  |  |
| Time of hearing deprivation | Years | 3.8 | 3.4 | 1.2 | 5.6 | 6.8 | 1.9 | 3.8 | 1.2 | 6.8 |
| Follow‒up | Years | 4.8 | 2 | ‒ | 1.9 | ‒ | 6.6 | 3.8 | 1.9 | 6.6 |
| CI usage time | Hours/day | 6.9 | 9.5 | 12.1 | 9.8 | ‒ | 5.6 | 8.8 | 5.6 | 12.1 |

^a^ Studies considered in the meta-analysis.
